# Identification of microRNA profiles in salivary adenoid cystic carcinoma cells during metastatic progression

**DOI:** 10.3892/ol.2014.1975

**Published:** 2014-03-14

**Authors:** WEI CHEN, XIAOGE ZHAO, ZHEN DONG, GANG CAO, SENLIN ZHANG

**Affiliations:** 1 Department of Stomatology, Nanjing Jinling Hospital, Nanjing University School of Medicine, Nanjing, Jiangsu 210002, P.R. China; 2 Key Laboratory of Environment and Genes Related to Diseases, Xi’an Jiaotong University College of Medicine, Xi’an, Shaanxi 710061, P.R. China

**Keywords:** microRNA profile, adenoid cystic carcinoma, metastasis

## Abstract

Salivary adenoid cystic carcinoma (SACC) is a common type of salivary gland cancer. The poor long-term prognosis of patients with SACC is primarily due to local recurrence, distant metastasis and perineural invasion. MicroRNAs (miRNAs) have been identified as important post-transcriptional regulators, which are involved in various biological processes. The aim of the present study was to identify the miRNA expression profiles that are involved in the metastatic progression of SACC. Therefore, microarray technology was employed to identify miRNA expression profiles in an SACC cell line, ACC-2, and a highly metastatic SACC cell line, ACC-M, which was screened from ACC-2 by a combination of *in vivo* selection and cloning *in vitro*. Differences in miRNA expression were assessed by quantitative polymerase chain reaction (qPCR) assay. In addition, the potential target genes that are regulated by selected miRNAs were analyzed by various target prediction tools. The microarray data revealed that the levels of 38 miRNAs significantly differed between the ACC-M cells and the control ACC-2 cells. Six miRNAs (miR-4487, -4430, -486-3p, -5191, -3131 and -211-3p) were selected to validate the microarray data via qPCR. The expression of two miRNAs (miR-4487 and -4430) was significantly upregulated in the ACC-M cells, while the expression of two other miRNAs (miR-5191 and -3131) was significantly downregulated in the ACC-M cells. The potential target genes that were identified to be controlled by the six selected miRNAs were divided into four groups according to function, as follows: Apoptosis and proliferation (46 genes), cell cycle (30 genes), DNA damage and repair (24 genes) and signaling pathway (30 genes). The identification of microRNA expression profiles in highly metastatic SACC cells may provide an improved understanding of the mechanisms involved in metastatic progression, which would aid in the development of novel strategies for the treatment of SACC.

## Introduction

Salivary adenoid cystic carcinoma (SACC) is a frequent subtype of salivary gland malignancy accounting for 25% of malignant tumors in the major salivary glands (1) and 50% in the minor glands (2). The neoplasm is characterized by heterogeneous phenotypic features and persistently progressive biological behavior. Although various treatment options have been extensively investigated, the poor long-term prognosis for patients with SACC is primarily due to local recurrence associated with perineural invasion (PNI) and delayed onset of distant metastasis, particularly to the lungs (3,4).

Therefore, it is necessary to identify and understand the diverse mechanisms behind metastasis so as to improve the treatment strategies for SACC patients. Genomic and proteomic studies have yielded various novel insights into molecular targets and the mechanisms of SACC metastasis (5,6). A number of mechanisms underlying the distant metastasis of SACC have been described, including the Notch gene family, matrix metalloproteinases, nerve growth factor, and vascular endothelial growth factor (7–12). However, the mechanisms of distant metastasis are complicated and remain obscure; therefore, further elucidation is required.

The changes in gene expression that accompany metastasis include DNA, mRNA and protein levels, however, the changes in the levels of mRNA and the encoded proteins are often disproportionate. This may be due to a number of reasons, among which post-transcriptional regulation by microRNAs (miRNAs) is particularly important. miRNAs are small non-coding RNAs of 21–25 nt that negatively modulate protein expression (13). miRNAs are involved in various biological processes, including development, proliferation, apoptosis and differentiation (14,15). In addition, studies have demonstrated that aberrant microRNA expression is correlated with malignant transformation and tumor development (16,17).

Thus, it was hypothesized that miRNAs may be significant in mediating metastatic progression in SACC. In order to investigate this hypothesis, a microRNA array was employed to detect a distinctive miRNA expression pattern between an SACC cell line, ACC-2, and a highly metastatic SACC cell line, ACC-M, which was screened from ACC-2 by a combination of *in vivo* selection and cloning *in vitro* (18). Since ACC-2 and ACC-M cells have an identical genetic background (with the exception of different metastatic behavior), it was presumed that differentially expressed miRNAs were metastasis-related miRNAs, which are involved directly or indirectly in the progression of metastasis. The results of the miRNA microarray analysis were further verified by quantitative polymerase chain reaction (qPCR). In addition, bioinformatic methods were employed in the analysis of the miRNA expression arrays to identify the target genes, which are regulated by miRNAs, and analyze the gene functions that are associated with the metastasis of tumors. The miRNA signature of metastatic progression may aid with the development of novel therapeutic strategies for SACC patients.

## Materials and methods

### Cell lines and cell culture condition

The cell lines, ACC-2 and ACC-M, were provided by the Department of Oral Biology, School of Stomatology, Fourth Military Medical University (Xi’an, China). The two types of cells were cultured in Dulbecco’s modified Eagle’s medium (Life Technologies, Carlsbad, CA, USA) supplemented with 10% fetal bovine serum (Life Technologies), 2.05 mM L-glutamine, 100 *μ*g/ml penicillin and 100 *μ*g/ml streptomycin at 37°C with 5% CO_2_.

### miRNA microarray

Total RNA from the SACC cell line, ACC-2 and the highly metastatic SACC cell line, ACC-M was isolated using TRIzol reagent (Life Technologies). The quality and quantity of the RNA samples was assessed by standard electrophoresis and ultraviolet (UV) spectrophotometry methods (19). The miRNA microarray analysis was performed using the Human miRNA OneArray v4 (Phalanx Biotech Group, Hsinchu, Taiwan), which contains 1,884 unique microRNA probes that are complementary to human microRNA sequences (Sanger miRBase Release 18.0). The data from the microarray were collected and analyzed in accordance with the Minimum Information About a Microarray Experiment guidelines (20). The differential miRNA expression was determined using a two-tailed t-test on a single miRNA basis. The signals were considered to be different when P<0.01 and these miRNAs were subsequently selected for cluster analysis. The miRNA microarray analyses were performed in duplicate and the miRNAs that exhibited common differential expression levels were selected.

### qPCR analysis of miRNA expression

qPCR assays were performed on two samples. Using the TaqMan MicroRNA assay kit (Life Technologies), which utilizes miRNA-specific primers, each RNA sample (10 *μ*g) was reverse transcribed according to the manufacturer’s instructions. The resulting cDNA was semi-quantitatively amplified in 45 cycles on an ABI 7500 Real-Time PCR system, using TaqMan^®^ Universal PCR Master Mix, No AmpErase^®^ UNG and TaqMan MicroRNA assays for hsa-miR-4487, hsa-miR-4430, hsa-miR-486-3p, hsa-miR-5191, hsa-miR-3131, hsa-miR-211-3p and U6 snRNA (all Applied Biosystems, Carlsbad, CA, USA). These miRNAs were selected as they exhibited the greatest fold change (hsa-miR-4487 and hsa-miR-4430 were upregulated, and hsa-miR-5191 and hsa-miR-3131 were downregulated), or were of potential importance as indicated by previous studies (hsa-miR-486-3p was upregulated and hsa-miR-211-3p was downregulated). Each qPCR assay was performed at least three times (21,22).

### miRNA target prediction

Candidate miRNAs that were selected for the qPCR analysis were utilized for target prediction. The potential miRNA targets among the genes, which were negatively correlated with miRNA expression, were determined using the publicly available TargetScan (http://www.targetscan.org), miRBase (http://www.ebi.ac.uk/enright-srv/microcosm/htdocs/targets/v5) and PicTar (http://pictar.mdc-berlin.de) algorithms. The genes that were predicted as candidate miRNA targets and those that were selected on the basis of Gene Ontology (http://www.geneontology.org) were compared, and the genes that appeared in the two lists were included in the present study.

### Statistical analysis

Statistical analysis was performed using SPSS 17.0 statistical software (SPSS, Inc., Chicago, IL, USA). All numerical data from the qPCR assays were analyzed following the derivation of standard curves for each miRNA of interest. Statistical analysis was performed with Student’s t-test and P<0.05 was considered to indicate a statistically significant difference. Each experiment was performed in triplicate and each individual sample was run in triplicate.

## Results

### Analysis of the quality of RNA isolated from ACC-2 and ACC-M cells

**Figure 1:**
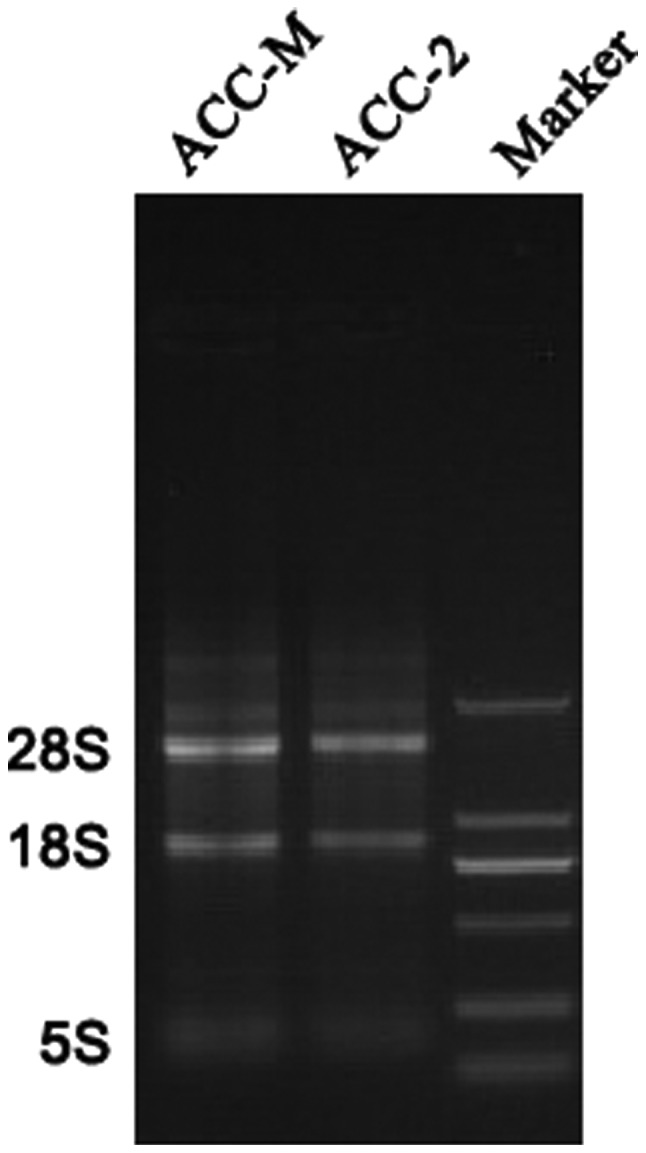
Denaturing agarose gel electrophoresis of RNA isolated from the cell samples. There are three clear bands (28S, 18S and 5S). The brightness of the 28S band was approximately twice that of the 18S bank, suggestive of high quality RNA. ACC-M was screened from ACC-2 by a combination of *in vivo* selection and *in vitro* cloning. The size of marker bands are 100, 250, 500, 750, 1,000 and 2,000bp from the bottom to top.

The quality of total RNA was analyzed via denaturing agarose gel electrophoresis and UV spectrophotometry (UV-2600; Shimadzu Corporation, Kyoto, Japan). RNA that was isolated from the ACC-2 and ACC-M cells exhibited clear bands of 28S rRNA and 18S rRNA (Fig. 1). The OD_260_/OD_280_ values of each sample were 2.05 and 2.06, respectively, and the OD_260_/OD_230_ values of each sample were 2.05 and 2.17, respectively, as determined by the UV spectrophotometer. The RNA quality was confirmed by the Agilent 2100 Bioanalyzer (Agilent Technologies, Santa Clara, CA, USA) prior to further hybridization. All these results indicated that the obtained high quality RNA was suitable for microRNA microarray analysis and the following qPCR analysis to verify the results of the microarray.

### miRNA expression profiles in the SACC cell line, ACC-2 and the highly metastatic SACC cell line, ACC-M

To identify the changes in the miRNA expression profiles between the SACC cell line, ACC-2 and the highly metastatic SACC cell line, ACC-M, miRNA microarray analysis was conducted. The miRNA expression profile of the two SACC cell lines revealed that 38 out of 1,884 human miRNAs were differentially expressed between the ACC-2 and ACC-M cell lines. Of the 38 miRNAs identified as differentially expressed, 20 were upregulated (Table I) and 18 were downregulated (Table II) in the ACC-M cell line compared with the control ACC-2 cell line. In the average fold-change analysis, 17 of the 38 flagged miRNAs exhibited >2-fold change in expression level, while there were 21 miRNAs that exhibited <2-fold change in expression level (Table I and II).

### Confirmation of miRNA microarray data by qPCR

**Figure 2:**
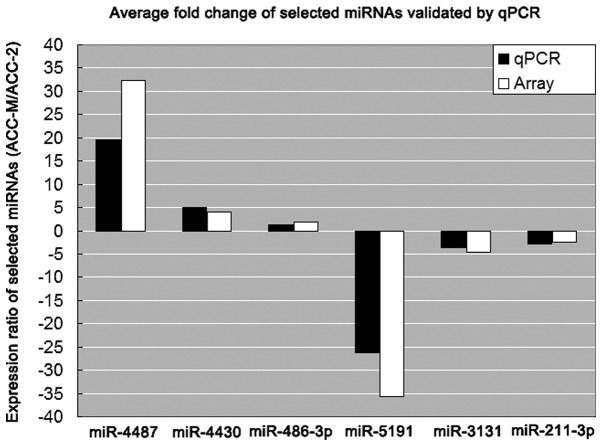
Validation of microarray analysis data by qPCR for candidate miRNAs associated with metastatic progression of ACC-M cells. The expression levels of six miRNAs (miR-4487, -4430, -486-3p, -5191, -3131 and -211-3p) were determined by qPCR. Each qPCR assay was performed at least three times. miRNA, microRNA; qPCR, quantitative polymerase chain reaction.

To confirm the miRNA microarray data, a TaqMan MicroRNA assay kit was used to perform qPCR analyses of the expression levels of miR-4487, -4430, -486-3p, -5191, -3131 and -211-3p in the ACC-2 and ACC-M cell lines. It is generally accepted that gene expression levels should be normalized by a carefully selected, stable internal control gene. To validate the assumption of stable expression of a given control gene, prior knowledge of a reliable measure to normalize this gene, in order to eliminate any non-specific variation, is required. For each sample, the expression values were normalized to the U6 snRNA gene (a stable housekeeping gene) and the expression levels relative to the ACC-2 cell line were calculated. The expression pattern that was identified in the arrays was confirmed by this additional analysis. Compared with the ACC-2 cells, three miRNAs (miR-4487, -4430 and -486-3p) were significantly upregulated, while three miRNAs (miR-5191, -3131 and -211-3p) were significantly downregulated in the highly metastatic SACC cell line, ACC-M. For these candidate miRNAs, the qPCR analysis revealed similar patterns of up- or downregulation to those revealed by the results of the microarray analysis (Table III; Fig. 2), although the magnitude of the reported changes in expression differed. Thus, miRNA expression profiles clearly exhibit significant differences between ACC-2 and ACC-M, indicating that miRNAs are significant during the metastatic progression of SACC.

### Prediction of miRNA targets

The identification of potential target genes of miRNAs that are associated with the metastatic progression of SACC is essential to investigate their biological functions. Previous studies have shown that miRNAs are able to regulate expression of their target genes by decreasing mRNA stability in addition to translational inhibition (23). The candidate miRNAs, which were used for qPCR, were selected for target prediction and analyzed with the following three target prediction tools: TargetScan 6.2 (Whitehead Institute for Biomedical Research website; http://www.targetscan.org); miRBase Targets Release v5 (the Enright Group; http://www.ebi.ac.uk/enright-srv/microcosm/htdocs/targets/v5); and PicTar (http://pictar.mdc-berlin.de). By analysis of these databases, human genes, which were known to be involved in cell proliferation, apoptosis, cell cycle, DNA damage, DNA repair and signaling pathways were selected from the Gene Ontology website (http://www.ebi.ac.uk/GOA). Those genes that were predicted to be targets of the candidate miRNAs were selected and are listed in Table IV. These data may provide the foundation for further analysis of the mechanisms of metastatic progression in SACC.

## Discussion

SACC is a common malignant tumor that arises from the secretory epithelial cells in the salivary glands of the head and neck. SACC accounts for 1% of all head and neck malignancies and 10% of all salivary neoplasms (24). Although SACC tend to grow slowly, this neoplasm has a poor prognosis owing to its insidious invasion into adjacent tissues and hematogenous spread to distant organs (lungs, bone and liver) (24,25). The five-year disease-free survival rate is ≤90%, however, this reduces to 40% after 15 years (27). Advanced tumor stage, distant metastasis to the lungs, PNI, and solid subtype are among the clinicopathological parameters that have been proposed as useful predictors of the clinical course of SACC (28). However, accurate prediction is often challenging, and in-depth studies on invasion and metastatic mechanisms are of great significance for prognosis and evaluation, and the selection of appropriate treatment protocols.

miRNAs are short non-coding RNAs of 21–25 nt that regulate gene expression in multicellular organisms. Numerous studies have indicated that miRNAs are significant in carcinogenesis and tumor progression in certain types of cancer, including SACC (29–31). However, to the best of our knowledge, the altered expression of miRNAs, which is associated with metastatic progression in SACC, has not been investigated. Therefore, in the present study, high-throughput miRNA microarray technology, with 1,884 miRNAs selected from the miRBase, was used to detect miRNA expression in parental ACC-2 cells and highly metastatic ACC-M cells. The microarray data revealed that 38 miRNAs exhibited significant differences in expression between ACC-2 and ACC-M cell lines; 20 were upregulated and 18 were downregulated in the ACC-M cells. In the present analysis, 17 miRNAs in the ACC-M cells exhibited >2-fold changes compared with the control cells (ACC-2). Although not all miRNAs in ACC-M cells exhibit similar regulation patterns, numerous miRNAs with ≥2-fold expression changes were identified in the present study for further investigation. Therefore, to confirm the expression of the six miRNAs that were identified in the microarray analysis, a qPCR assay was performed. The results of the qPCR analysis of the upregulated (miR-4487, -4430 and -486-3p) and downregulated (miR-5191, -3131 and -211-3p) miRNAs are demonstrated in Fig. 2. For all six miRNAs, the qPCR experiment confirmed their down- or upregulation, however, the magnitude of the changes in the expression levels was found to differ between the microarray and qPCR analyses.

Thus far, to the best of our knowledge, there have been no reports of a correlation between the six miRNAs discussed in the present study and the metastatic progression of SACC. To elucidate the regulatory mechanisms of those miRNAs, which are associated with the metastatic progression of SACC, various bioinformatic methods were employed to identify potential target genes of those miRNAs. The target genes, which have been reported to be associated with the metastatic progression of human tumors, were divided into four groups according to function (as described by the Gene Ontology website, http://www.geneontology.org): Apoptosis and proliferation (46 genes); cell cycle (30 genes); DNA damage and repair (24 genes); and signaling pathway (30 genes; Table IV).

Computational predictions indicate that one miRNA is able to target numerous mRNAs and each mRNA can also be targeted by numerous miRNAs. Thus, a one-to-one association between miRNAs and target genes should not be expected, rather a one-to-many or many-to-one association. Previous studies have demonstrated that functional miRNA binding sites can lie in the coding or 5′-untranslated regions of endogenous mRNAs (31). Therefore, the number of target genes that are regulated by miRNAs may be larger than what was identified by the bioinformatic methods used in the present study. Although the expression levels of miRNAs may demonstrate inverse correlations with the protein expression levels of their potential target genes, further studies are required to confirm a regulatory association between miRNAs and the predicted target genes.

In conclusion, SACC is a common subtype of salivary gland malignancy, which exhibits biological behavior that facilitates hematogenous spread to lungs. In the present study, the experimental data demonstrated that alteration of the miRNA expression profile in the ACC-M cell line may be associated with metastatic progression. These findings may aid with elucidating the potential mechanisms that underlie the metastatic progression of SACC and provide a novel molecular therapeutic target for the treatment of SACC.

## Figures and Tables

**Table I:** miRNAs upregulated in the ACC-M cell line compared with the control ACC-2 cell line.

Phalanx ID	Name	Average fold change
PH_mr_0004751	hsa-miR-4487^a^	32.24
PH_mr_0004716	hsa-miR-4430^a^	4.05
PH_mr_0004876	hsa-miR-5096^a^	3.61
PH_mr_0000840	hsa-miR-1285-3p^a^	2.85
PH_mr_0008030	hsa-miR-1273g-3p^a^	2.60
PH_mr_0004900	hsa-miR-3150b-3p^a^	2.59
PH_mr_0004874	hsa-miR-1273f^a^	2.46
PH_mr_0001931	hsa-miR-1273a^a^	2.43
PH_mr_0004658	hsa-miR-1273e^a^	2.25
PH_mr_0000379	hsa-miR-574-5p	1.98
PH_mr_0004873	hsa-miR-5095	1.88
PH_mr_0004549	hsa-miR-4638-5p	1.88
PH_mr_0004565	hsa-miR-4688	1.87
PH_mr_0001461	hsa-miR-877-5p	1.86
PH_mr_0004257	hsa-miR-3154	1.82
PH_mr_0003162	hsa-miR-766-3p	1.80
PH_mr_0004018	hsa-miR-1972	1.78
PH_mr_0000953	hsa-miR-486-3p	1.78
PH_mr_0004577	hsa-miR-4728-5p	1.74
PH_mr_0004187	hsa-miR-1976	1.74

a miRNAs exhibiting >2-fold expression change compared with ACC-2.

miRNA, microRNA.

**Table II:** miRNAs downregulated in the ACC-M cell line compared with the control ACC-2 cell line.

Phalanx ID	Name	Average fold change
PH_mr_0008120	hsa-miR-5191^a^	0.028
PH_mr_0004035	hsa-miR-3131^a^	0.22
PH_mr_0004331	hsa-miR-4278^a^	0.26
PH_mr_0004534	hsa-miR-4498^a^	0.39
PH_mr_0008014	hsa-miR-211-3p^a^	0.40
PH_mr_0004731	hsa-miR-4450^a^	0.41
PH_mr_0000102	hsa-miR-373-5p^a^	0.44
PH_mr_0000010	hsa-miR-7-5p^a^	0.48
PH_mr_0008044	hsa-miR-5010-5p	0.52
PH_mr_0004714	hsa-miR-4428	0.53
PH_mr_0001673	hsa-miR-18b-5p	0.53
PH_mr_0001728	hsa-miR-18a-5p	0.53
PH_mr_0004543	hsa-miR-1587	0.54
PH_mr_0001696	hsa-miR-20a-5p	0.55
PH_mr_0000136	hsa-miR-1265	0.55
PH_mr_0000436	hsa-miR-92a-3p	0.56
PH_mr_0000747	hsa-miR-186-5p	0.57
PH_mr_0000812	hsa-miR-92b-3p	0.57

a miRNAs exhibiting less than half of the expression levels of ACC-2 cells.

miRNA, microRNA.

**Table III:** Validation of microarray analysis data by qPCR for candidate microRNAs.

		Average expression ratio	
		
Phalanx ID	Name	qPCR	Array
PH_mr_0004751	hsa-miR-4487	19.560	32.240
PH_mr_0004716	hsa-miR-4430	4.960	4.050
PH_mr_0000953	hsa-miR-486-3p	1.210	1.780
PH_mr_0008120	hsa-miR-5191	0.038	0.028
PH_mr_0004035	hsa-miR-3131	0.280	0.220
PH_mr_0008014	hsa-miR-211-3p	0.340	0.400

All data were confirmed by qPCR. qPCR, quantitative polymerase chain reaction.

**Table IV:** Predicted target genes of miRNAs.

	Putative targets of miRNAs according to function			
	
miRNA name	Apoptosis and proliferation	Cell cycle	DNA damage and repair	Signaling pathways
hsa-miR-4487	GIPC3, URGCP, ITGA5, VASH1, FGD1, HECA, FLT1	GALNTL2, NAP1L5, ZNF740, TBC1D4, CDKN1A	PSMA5, HIPK2, USH1G, BOLL	EFNB3, SORT1, RORC, FMOD, FAM123C, CUL3, CXCR3, WNT4
hsa-miR-4430	MAP3K9, MAP2K7, SHROOM4, KRT85, MNT, GLI2, IREB2	SEZ6, GRIN3A, MOCS1, PARP16	POMT2, RPP14, ZDHHC22	POP4, EIF2C1, POFUT1, EFNB3, CD72, LPHN2, EIF2C3
hsa-miR-486-3p	NAT15, GDI1, DMBX1, CNP, PAK6, CD276, MAP4, VASH1, PLA2G6, SPTBN4, SAP30BP, COL4A2, HS6ST1, DCAF7, TANC2	SF3A1, ATXN7L3, SDC3, TTYH3, STX1B, GATAD2B	FYCO1, STIM1, CACNA2D2	FLOT2, CTNNBIP1, MARK2, MAPKBP1, LOC388630
hsa-miR-5191	FOXL1, AHI1, PERP	MAP1B, CDH12, ABCA9, WWP2, HOXC6, EIF4E, CD1D	PPEF2, ZNF547, RANBP10, FLG2, NIPA2, PARP9, ZNF32, ZFP28	RBPJL, GNB1, GNB4, SOX9, YWHAZ, NOTCH2
hsa-miR-3131	SEMA6C, MEF2D, TMEM119, TRAF3, EIF4G1	RIMBP2, SYN1, FBXO22	NFIX	PPARGC1B
hsa-miR-211-3p	NFASC, SEMA5A, DMC1, MAFG, PITPNA, ERLIN2, PDXK, SMARCD1, SOCS5, TNFAIP1	MAU2, PDS5A, NOS1, ATP11A, XPO4	SMCR7L, ZNF217, OBFC1, RBFOX2, ATG10	PIK3CG, LONP2, PPP2R5E

miRNA, microRNA.

## Abbreviations

SACC: salivary adenoid cystic carcinoma
miRNA: microRNA
PNI: perineural invasion
qPCR: quantitative PCR
